# Parenting in the Face of Trauma: Music Therapy to Support Parent–Child Dyads Affected by War and Displacement

**DOI:** 10.3390/children11101269

**Published:** 2024-10-20

**Authors:** Tamar Hadar

**Affiliations:** School of Creative Arts Therapies, Faculty of Social Welfare and Health Sciences, University of Haifa, Haifa 3498838, Israel; thadar1@staff.haifa.ac.il

**Keywords:** music therapy, Child-Parent Psychotherapy (CPP), dyadic therapy, trauma, interpretative phenomenology

## Abstract

Background: The literature highlights the profound psychological impact of war on children, families, and communities, emphasizing the prevalence of Post-Traumatic Stress Disorder (PTSD), anxiety, and other symptoms among affected individuals. Interventions, such as Child-Parent Psychotherapy (CPP) and music therapy, show promise in mitigating trauma effects, underscoring the need for holistic approaches that address familial and community dynamics alongside individual well-being. Methods: Aiming to explore the influences of dyadic music therapy sessions on parents’ capacity to support their children, this study involved four families displaced from their home-kibbutz as result of a terrorist attack. All dyads participated in music therapy sessions with a focus on parent–child interactions and trauma processing (CPP informed). Embedded in a qualitative, phenomenological approach, the research utilized interpretative phenomenological analysis (IPA) and micro-analytic methods to explore meaningful moments in the music therapy sessions. Results: Findings identified four central categories: (1) Discovering the child’s grounding song: identifying resources; (2) Musical improvisation sets the grounds for parent–child mutual recognition of the child’s traumatic experience; (3) Musical performance empowers child and parent; (4) A sense of agency is gained through controlling the musical environment. Conclusions: The significance of restoring the children’s freedom of play, the parents’ sense of competency, and of enhancing families’ capacity to connect to their traumatic experiences through the musical environment is discussed.

## 1. Introduction

### 1.1. Children Experiencing War and Displacement

A significant body of literature highlights the psychological trauma and post-traumatic stress experienced by children who have been exposed to war [1,2,3,4,5]. Children living in chronic war zones display high rates of Post-Traumatic Stress Disorder (PTSD), anxiety, and other psychological symptoms due to continual exposure to violence and distressing events [6,7,8]. According to Sagi-Schwartz [7], the impact of war on children is mediated by a combination of risk and protective factors. Factors such as the availability of social support systems, familial relationships, and community cohesiveness play crucial roles in determining the extent of war’s debilitating effects on children. The research underscores the importance of understanding these dynamics for creating interventions that foster resilience among children [7].

Pat-Horenczyk et al. [9] found high levels of post-traumatic stress among both children and mothers in Sderot, Israel, due to frequent missile attacks, showcasing the wide-reaching psychological impact of constant conflict exposure on family units. The authors presented an integrative model focusing on clinical services, resilience-building workshops, and enhancing local resources in the community. The model was implemented effectively to address the symptoms and enhance resilience among affected families and educators. Preliminary results showed positive outcomes in reducing symptoms of distress in children and improving coping skills among parents and teachers, highlighting the potential of community and clinical efforts in fostering resilience in conflict-affected populations.

Moreover, the psychosocial consequences of war seem to extend beyond the individual level, to affect family and community systems [9,10]. Studies from Afghanistan and Sri Lanka [3] have established correlations between exposure to war and increased incidents of domestic violence. This interaction suggests the necessity for family- and community-based programs tailored to address the multifaceted impacts of mass trauma. Despite the profound adversities faced, some children develop resilience and find ways to cope with the horrifying experiences.

Bryant et al. [10] revealed the high rates of PTSD among refugee caregivers and its impact on child mental health. The researchers found that 38% of 411 adult caregivers who participated in their study had probable PTSD, which is consistent with other research on refugee populations. The study showed that caregiver PTSD persisted over time and was linked to harsh parenting, which negatively affected children’s mental health. This effect was stronger than the direct impact of PTSD on children’s emotional problems. The study also highlighted that previous trauma and post-migration stressors contributed to ongoing PTSD and harsh parenting, leading to various psychological issues in children. Understanding the impact of early and overwhelming stress on individuals is crucial because childhood trauma can predispose them to being overwhelmed by stress later in life and may lead to misdiagnosis of stress-related behaviors as conditions like ADHD [11,12]. The influence of trauma and war-induced trauma on parenting practices has been of growing interest in the past decade [10,13,14] and will be explored next.

### 1.2. Parenting, War and Intergenerational Trauma

The significance of parenting practices as either mitigating or exacerbating the impact of mass trauma on child adjustment was highlighted by different studies [9,10,13,14,15,16,17]. Khamis [18] showed how parental psychological distress served as a mediator in the impact of war on children’s emotional and behavioral disorders. Using hierarchal regression analyses, the study demonstrated how trauma exposure and parents’ psychological distress predicted the occurrence of disorders in children. In a study focusing on the long-term effect of the 9/11 attacks [19], the authors highlighted not only its influence on parents but the severe psychological effects on their children. In an attempt to mitigate the intergenerational transmission of trauma effects, the authors underscored the child’s risk of psychological distress when exposed to a combination of maternal depression and PTSD. This research contributes to the understanding of how very young children, given their developmental stage, are particularly vulnerable to the effects of maternal psychological conditions triggered by extremely stressful events. Additionally, it highlights the necessity of considering the mother–child dyad in the context of trauma exposure and treatment.

An important consequence of war relates not only to parental psychological distress and post-traumatic reactions but to its impact on their parenting practices [14]. Drawing from a wide range of studies, Murphy et al. [14] explored the complex dynamics of family relationships amidst the backdrop of war and explored how war related experiences impacts not only the mental health of parents but also affects their parenting styles, which in turn, influences the psychological and emotional well-being of children. The authors argued that parents exposed to war experiences may undergo significant shifts in their behavior and parenting styles. Such experiences can lead to increased authoritarianism, hesitancy, and withdrawal in how parents interact with their children. Conversely, some mothers, upon facing horrifying events, may become less strict and exercise lower control, thereby affecting the dynamics of mother–child interaction. Ultimately, this study emphasizes the intricate link between war events, parenting, and children’s mental health. It advocates for a holistic approach to intervention that considers the entire family system, aiming to mitigate the adverse effects of war trauma across generations. One such holistic, evidence-based and integrative approach to treatment of trauma-induced symptoms is Child-Parent Psychotherapy (CPP).

### 1.3. Child-Parent Psychotherapy

Child-Parent Psychotherapy is a trauma-informed dyadic intervention model for young children. This model is highlighted as an evidence-based intervention suited for the experiences of children, aiming to restore the child’s healthy developmental trajectory following traumatic experiences [20]. In a study examining the efficacy of Child-Parent Psychotherapy (CPP) in reducing symptoms of trauma in both children and their parents who have experienced trauma [21], the authors found a decrease in post-traumatic stress symptomatology (PTSS), indicating that CPP effectively ameliorates trauma symptoms in both population groups. Recent years gave rise to the integration of various art therapy approaches, among them music therapy, within trauma-informed practices [22,23]. We will now examine the distinctive aspects of music therapy that make it an appropriate clinical practice for clients who have experienced traumatic and post-traumatic symptoms.

### 1.4. Music Therapy in the Context of Trauma

A growing amount of literature delves into the multifaceted role of music therapy in addressing the complex needs of individuals experiencing trauma [2,24,25], including those bearing the weight of transgenerational trauma [26]. Abrams delineates an intimate exploration into how analytical music therapy (AMT) can be harnessed as a therapeutic conduit to navigate and alleviate the deeply ingrained scars of transgenerational trauma. Through reflective vignettes, the paper underscores AMT’s potency in facilitating a unique therapeutic space, enabling individuals to confront and therapeutically engage with transgenerational trauma. Several mechanisms underlying musical experiences are associated with the therapeutic effect of music therapy, and are pertinent to trauma-informed practice: relationality in music [24,27]; music-related emotional regulation [25,26,27,28,29,30,31]; and communicative musicality [32]. Bensimon [24] expands on the realms of relational needs (RNs) of trauma victims—within a spectrum encompassing recognition, acceptance, emotional witnessing, responsiveness, safety, trust, and outreach, the author elucidates how these pivotal psycho-emotional requirements can be efficaciously addressed within the music therapy milieu. Employing a constructivist methodology, the paper articulates the observed therapeutic mechanism whereby music validates, witnesses, and tactfully engages with the emotional states of trauma victims. Through music therapy, a profound space of emotional safety and understanding is crafted.

Several researchers advocate for the possible role of music intervention in supporting emotional regulation (ER) among clients [29,30,31]. Sena-Moore and Hanson-Abromeit [31] provide a theoretical rationale for the Therapeutic Function of Music (TFM) Plan, applied to a music-based emotional regulation intervention for preschoolers, known as the Musical Contour Regulation Facilitation (MCRF) intervention. The study suggests that music can effectively address limitations in current verbal and behavioral ER treatments by offering real-time, adult-child interactions that support stress management and emotional regulation through alternating arousal states. As trauma and post-trauma are highly associated with difficulties in emotional regulation [33,34], music-based interventions may be of particular interest when establishing parent–child, trauma-informed protocols.

Another aspect of the music therapy relevant to trauma processing is its non-verbal nature [23,32,35]. As described by Trevarthen and Malloch [32], music can be seen as a basic language that nurtures coordinated interaction and closeness, forming the basis for later verbal communication. When discussing the mechanisms of change underlying music therapy interventions in the treatment of trauma and post-trauma, Bensimon [23] highlights that music, as a sensory stimulus, can bypass linguistic barriers and access unprocessed traumatic memories, allowing clients to express trauma-related super-expressive emotions. Through the process of emotional contagion, the music therapist and client engage in a reciprocal exchange, where the therapist validates the client’s emotions, helping to restore empathy and enhance the therapeutic process. The benefit of employing a non-verbal approach for trauma processing becomes even more significant when working with children, as they may find verbal communication difficult or inaccessible as a result of such experiences [36].

### 1.5. The Current Research

A smaller range of literature deals with the impact of music therapy interventions on childhood and adolescents dealing with the consequences of stressful events (e.g., [2,37]) and even less attention is given to interventions including parents’ role in the context of trauma which, when addressed, is predominantly concerned with NICU-induced trauma [38,39,40]. Music and music therapy approaches have been used to address the needs of adults [25,41] and children [42,43,44] experiencing war and conflict-induced anxiety and trauma. Inasmuch, there is as yet a dearth of research concerning the significance of including parents in the therapeutic process, in the context of childhood and adolescence trauma. This study aims at shedding some light on the significance of including the child–parent dyad in therapy when dealing with war-induced trauma in a music therapy context. A more integral, holistic approach to childhood trauma, including the role of parents in mediating the traumatic experience and its by-products specifically in the context of war, is missing in the music therapy literature. This study aims at addressing this gap in the literature, by presenting a thematic analysis of four courses of dyadic music therapy provided to families who had experienced displacement for six months, following the deadly attack performed by Hamas on 7 October 2023.

Beyond presenting a music-based approach towards trauma, the current study introduces an ecological and psychobiological approach to therapy, through acknowledging different aspects of the child’s being, possibly affected by the distressing experience: the war induced trauma component (i.e., environmental and neural aspect); trauma’s possible influence on the child’s relations with their care-takers with consideration of child’s developmental stage and attachment predisposition [36]; trauma’s possible influence on the whole family system; and trauma’s influence on the community system as well as on the child’s specific peer-group. Throughout the analysis and interpretation of therapy vignettes, the author integrates multiple angles to encompass the various layers of well-being attended through the musical-therapeutic intervention, including the child’s changes in the physiological, behavioral, emotional, social, relational, and cognitive realms. Therefore, the author believes that, though originating in a specific, music-based mind-set, this paper can expand clinical thought in new integrative and trans-modal directions when addressing child and youth trauma.

### 1.6. Research Questions

The research questions guiding this analysis were:

1. What are the salient characteristics of significant moments in parent–child music therapy sessions for dyads who had experienced acute trauma?
2. How can those significant moments be understood in the context of each client’s individual course of therapy?
3. How can those significant moments be understood in the context of each client’s relationships with their parents?
4. How can those significant moments be understood in light of clients’ and families’ traumatic narratives?

## 2. Materials and Methods

### 2.1. Background and Intervention

The participants in this study included four families who were displaced from their home-kibbutz situated on the Israeli border with Gaza, following Hamas’s deadly terror attack on 7 October 2023. The families were relocated to a hosting village in the northern part of Israel. As part of the governmental rehabilitation plan, all families were offered by social services the opportunity to meet with various professionals (e.g., social workers, psychologists and art therapists), to process their traumatic experiences and the consequent displacement, which endured for six months. The author is a music therapist who participated in this plan by initiating a temporary music therapy clinic in the hosting village, offering music therapy sessions for families of young children and adolescents. The author is an experienced music therapist, who also specialized in Child–Parent Psychotherapy.

Most of the families who engaged in the music therapy program were interested in dyadic, i.e., parent–child, meetings, which were encouraged by the therapist, who incorporated a CPP protocol into her approach. However, several families chose to engage only their child in therapy. All families met for parental consultations separately. All the children met for weekly one-hour sessions.

To foster inclusive language and perspectives, the author acknowledges the impact of her cultural background on social science inquiry ([45,46]). As an Israeli, white female studying music and music therapy in Western-oriented institutions, she recognizes the potential for oppressive language and thinking. While this study primarily examines the effects of war and trauma on a specific group affected by a terror attack, the author acknowledges the broader impact of war on all involved parties, hoping that the research findings will benefit anyone experiencing trauma. Throughout the paper, person-first language is used, such as “children who experienced trauma” rather than “traumatized children.”

### 2.2. Participants

Four participants were included in the analysis (see Table 1). The inclusion/exclusion criteria were as follows: (1) families who opted for Child-Parent Therapy instead of individual sessions for the child; (2) the child had no additional diagnoses other than experiencing the traumatic event of the October 7 attack; and (3) the dyad attended a minimum of four therapy sessions.

### 2.3. Research Approach

Embedded in a qualitative, phenomenological school [47,48], this study was informed by both interpretative phenomenological analysis (IPA) approaches in the study of music therapy sessions ([49,50,51]), as well as micro-analytic methods applied for the analysis of musical and non-musical occurrences within the client–therapist relationship [49,52,53]. Hadar and Amir [50] adapted Forinash and Gonzales’s phenomenological method to investigate moments of joint improvisation in music therapy, adapting their multi-stage process into a four-step analysis of meaningful moments. Lee and Mcferran [49] integrated IPA with video micro-analysis, highlighting both the possibility of studying delicate nuances emerging in every session, without leaving out their contextual relationship to the therapy as a whole.

### 2.4. Procedure

Informed mainly by Hadar and Amir [50] and Lee and McFerran [49], the following steps were applied: (1) Preliminary Data Screening—Congruent with Smith et al. [48], all data included (in this case: the recordings of the sessions) were reviewed thoroughly. The author listened to all recordings of all participants’ sessions and indicated moments which revealed themselves as possibly significant to the research foci: parenting and trauma. (2) Understanding the moment—similar to Lee and McFerran [49]’s approach, in this phase, a short narrative was developed around certain moments, which were deemed important and had been marked by the author in the first stage. (3) Understanding the whole—following Lee and McFerran [49], in this stage the moments were contextualized within the bigger picture of the child’s course of therapy as unfolded through the author’s immersion in the recordings. This step included a first level of meaning induction out of the raw data. (4) Contextualizing the moment in the triadic or dyadic relationships in the room—this part leans on Hadar and Amir [50], who emphasized listening to the relationship in the music in their phenomenological protocol. In this study, the researcher highlighted the possible relational meanings, including both the child–parent relationship, the therapist–child relationship, and the therapist–parent relationship. Although not always present in the room, the role of the parent and their relevance to the moment was highlighted. (5) Contextualizing the moment in the traumatic experience—all moments were revisited in light of the family’s traumatic story, and possible interpretations were applied by the therapist-researcher. (6) Integrating parts and whole—following both Hadar and Amir [50] and Lee and McFerran [49], this stage supported the therapist-researcher in consolidating overlapping descriptions from earlier stages into a unified narrative of meaningful dyadic (and triadic) moments. The process of iteratively analyzing parts and wholes directed the therapist-researcher in exploring layers of influences, relating to the trauma as well as to the child–parent relationship, until reaching personal saturation. In this step, the author deployed Corbin and Strauss’s (2008) [54] coding method as follows: first, the author used open coding to break down the data into discrete parts, and to examine and compare them for similarities and differences. This phase included developing categories from the narratives created for each client, by assigning labels to chunks of data, such as words, sentences, or paragraphs. These labels represented key concepts or themes emerging from the data. This step was led by the author’s research questions, focusing on the possible meanings ascribed to participants’ experiences in the sessions, in the context of their traumatic narratives and the relational dynamics. Reoccurring patterns across clients were highlighted, giving rise to codes such as “being in control”; “singing as grounding”; “the child as a director” and “trauma narratives”. In the next stage, axial coding was deployed to organize the open codes into more distinct categories and to systematically explore the relationships among them. This permitted the researchers to gather new data while also building on the categories that had already emerged. At this point, following Charmaz’s [55] approach, the research focus was further honed, allowing the researchers to formulate their theoretical framework. In this step the author further examined the multi-layered meanings of the emerging categories and continued to consolidate the thematic analysis. In this step, categories such as “grounding songs elevate regulation” and “musical recordings serve as agents of resilience and strength” were articulated. In the final stage of analysis, selective coding was used to develop the core categories explaining the data. In line with Charmaz [56], this step involved an iterative process, in which categories were compared and contrasted to refine them further. The final consolidation of the core categories constructed the findings of this study, presented in Section 3.

### 2.5. Ethical Considerations

The researcher did not have access to a formal ethics review committee when conducting this study. This study followed the principles of the Declaration of Helsinki. Informed consent was obtained from the research participants prior to their engagement in music therapy sessions. In addition, all names and identifying information were removed from the data. Another important aspect pertinent to the current study relates to the dual role of the music therapist, serving both as a practitioner and a researcher in the study. The advantage of this arrangement is ascribed to the researcher’s ability to bring a first-person perspective grounded in extensive clinical experience, which can enhance the depth of reflection, insight, and the meaningful interpretation of findings. In the current study, this dual position enabled the researcher to sound fragile moments of existence for families who had faced horrifying events, and to further make sense of their meeting together, in a different time and space.

However, it also highlights the challenges that come with this dual role, particularly the need to maintain transparency, ensure the research is transferable and accountable, and remain open to different perspectives. The researcher’s unique situatedness is also highly germane to the research process itself. It is important to be aware of the potential biases and dilemmas that can arise from this dual role [57]. To address this issue, the short narratives created from each course of therapy, as well as the final thematic analyses, were all sent to the families for their approval. All families approved the materials and felt that they reflected their experiences. One family asked to remove a single quote, which was immediately removed from the data. In addition, the final analysis was sent to four experts in the fields of trauma treatment and qualitative analysis of practice materials for peer debriefing [58], and their comments were integrated to the final manuscript.

## 3. Results

The analysis of the sessions of four children who came to music therapy with their parents gave rise to four categories: (1) Discovering the child’s grounding song: identifying resources; (2) Musical Improvisation sets the grounds for parent–child mutual recognition of the child’s traumatic experience; (3) Musical performance empowers child and parent; (4) Gaining a sense of agency through controlling the musical environment.

### 3.1. Discovering the Child’s Grounding Song: Identifying Resources

A major component of all four courses of therapy was manifested in the creation of a grounding-promoting song for each child. In most cases, the grounding song was created in the second or third session, and soon after became a safe base for the child to turn to whenever the meeting became too intense. Mostly, grounding songs were played at the openings and closures of sessions.

Upon her second session, Yana marched in and confessed—“Yesterday I felt free—almost like I felt in music with you, it was when I rode on a horse for the first time”. Soon after, Yana and the therapist improvised a song, which included a short melodic dialogue between them:

Yana—“Yesterday something happened when I rode the horses.”

Therapist—“What happened”?

Yana—“I was so happy…I was so thrilled.”

The refrain “I was so thrilled” was repeated by the therapist and Yana many times, accompanied by Yana’s free play on the drum-set, and the therapist’s improvising on the piano. Since its first appearance in therapy, the sessions consistently opened and concluded with this song.

In Rani’s case, it took several sessions to focus on a certain song. Rani came to most sessions with his dad. After singing through several songs, Rani asked to record one of the Jewish holiday songs. The choice of song, which seemed random at first, revealed itself as touching some basic needs of Rani relating to stability and familiarity. Throughout many sessions, Rani worked on the process of recording the song, adding layers of various instruments to it, for himself as well as his father, enjoying moments of togetherness and synchrony.

Sarit, a young and gentle singer-songwriter, 9 years of age, used music composition extensively, both to connect to areas of strength and resilience, as well as for touching on more turbulent areas of her emotional world. For a considerable part of her therapy time, Sarit started the sessions by playing together with the therapist two–three songs she had learned on her guitar, prior to her sudden leaving of her home due to the terror attack. As her relationship with the therapist developed, Sarit immersed herself in putting new words to the familiar tunes, at first connecting to her dreams and fantasies, and later also revealing her fears and challenges. Through the songs, for example, Sarit expressed her concern for family friends who were being held captive in Gaza by Hamas.

### 3.2. Musical Improvisation Sets the Grounds for Parent–Child Mutual Recognition of the Child’s Traumatic Experience

The analysis revealed that, often after moments of freely exploring the musical instruments or improvising together, the children related spontaneously to a previously unbearable moment, most often revealing a hidden, yet unspoken part of their traumatic story. (Music therapy improvisation is a multifaceted practice, portraying the intentional use of musical expression to address various therapeutic goals. Whether in a medical setting, working with neurological conditions, in social contexts, or delving into psychological and psychodynamic aspects, the therapist guides the improvisational process to tap into the client’s needs and experiences. It is this blend of spontaneity, creativity, and therapeutic intentionality that makes music therapy improvisation such a powerful tool for healing and growth ([59,60])) In Yana’s third session, she started by singing and improvising musically for several minutes around her grounding song, a song she often turned to at different points of the session to connect to her strengths and resources. At this time, after singing her grounding song, she grabbed the microphone and started narrating a minute-by-minute description of her 17-h experience of hiding in the house-shelter when the terrorists attacked. She was looking at mom in the eye and did not stop talking for about 20 minutes. Mom looked at me intermittently, amazed, somewhat shocked by the unexpected monologue taking place in the room. While I was sharing with her the almost sacred feelings of Yana’s words, I also reassured her that this moment was being recorded. One of the salient qualities captured in the recording relates to the “stand-up” tone used by Yana, and her connecting for the first time to details which were out of reach for her for several weeks (as indicated by the family later), while she was navigating between moments of horror…

“At first, I asked my dad—is this real? Am I only imagining this? But then, another siren came, and we ran to the shelter, and then I opened Tik-Tok and I saw shooting, constant shooting, 24/7.”

and moments which gave her strength in those long hours, for example, her mom going out of the shelter to prepare a feast meal from all the meat she had prepared to cook that day for the unattended family gathering.

Another client, Rani, when asked specifically about the horrifying moments of waiting to be rescued from the terror attack, expressed detached feelings towards the situation, sharing that he could only remember watching a lot of movies. However, several weeks into the therapeutic process, he could share the experience of a traumatic trigger when hearing a siren when playing in the park with his friends (already in the hosting village), his parents being a few hundred meters away, not in sight. The therapy started with Rani’s improvisation on the recorder, followed by a joint improvisation between his father, the therapist and Rani, led by his recorder playing. Soon after putting the recorder aside, Rani could connect for the first time to feelings of helplessness, anxiety and fear when remaining alone in a moment of horror, amidst a possible missile attack. “I would rather stay out-side when the missile comes, than to go on my own to the shelter”, he added, hence referencing for the first time the levels of panic involved the last time he had to rush to a shelter, on 7 October, a dread he could not imagine going through alone. In the following moments, we could touch for the first time on the more shattered parts of his experience of the 7 October attack.

### 3.3. Musical Performance Empowers Child and Parent

For all children, moments of listening to their recordings with the parent or performing “live” to the parents were marked as highly significant, and in many cases enabled moments of transformation in the child’s well-being, as well as in the child–parent relationship.

It was in the very early stages of therapy that Sarit discovered she could prepare a mini-album in therapy, including a special performance of all the songs to her parents. In our very last session, her parents came to the special concert she had prepared with the therapist. Sarit felt on top of the world. Her parents were in awe and could not stop expressing their amazement at their daughter. Never had they experienced her as such a confident and impressive musician. In their final parental consultation, the parents shared with the therapist their surprise at the level of depth expressed in her songs’ lyrics. They had not realized until that moment how deeply her thoughts and emotions were centered around her war experiences.

Rani’s grounding song entailed different aspects of his therapeutic growth, addressing various goals. One facet of his work relates to the meticulous attitude he embraced when recording multiple layers for the song, together with dad. Rani’s musical requests from dad when recording his song together, across many sessions, reflected in many cases, Rani’s basic needs: sometimes, he only wanted to be listened to, and at other times he could engage in an improvised musical dialogue. A special moment emerged when mom visited the session for the first time. Rani immediately wanted to add a recorded layer of her playing and chose for her a very special instrument. Upon listening together to mom’s recording, he realized he could not hear her clearly enough and continued to record her part again and again until satisfied. Reflecting together with mom on possible meanings of Rani’s request in the session itself and afterwards marked an important milestone in the dyad’s relationship.

### 3.4. Gaining a Sense of Agency through Controlling the Musical Environment

One salient characteristic of the musical behavior among all four children included in this report, relates to their attempt to gain a sense of control through directing the musical activities in the session. For Maya, restoring her sense of control became a central theme in her course of therapy. Already in her first meeting, Maya was preoccupied with directing mom and me in every single step we made in our joint music making. In fact, Maya was very concerned about communicating her detailed instructions about “what happens when”, leaving us very little time to actually play together, if at all. Another manifestation of Maya’s need for control took place in Maya’s recording of “the passing of time”. While many children who participate in music therapy enjoy recording their music and listening to it, Maya insisted on recording the silence, while making the therapist and her parent freeze any move (and sometimes breath) while she recorded it and, later, when listening to it. In a slow and delicate process, Maya started to loosen her need to control the situation and direct every step made in therapy and managed to start restoring her ability to play and explore freely.

As mentioned earlier, in his music therapy meetings, Rani created a multi-layered instrumentation for a song. This creative endeavor reflected on many psychological processes Rani was going through. For example, in each layer, Rani added more instruments, changing the musical roles adopted by him and his dad, changing their sounds and their way of relating musically. While, in the first layer, Rani, the therapist and dad were asked by Rani to play and sing the song while adhering strictly to the script (words and music), in the second layer more musical freedom was added, yet dictated closely by Rani. Through articulating the delicate facets of each layer, Rani enabled himself to gain more levels of freedom, in his inner self, as well as within his relationship with his parents.

## 4. Discussion

The analysis identified four central categories highlighting different aspects of the child–parent relationship in the context of their traumatic experience, as manifested in music therapy. Through integrating different ways in which the war affected the children’s sense of safety and attachment, aspects relating to the opportunity music therapy provided for restoring salient child–parent properties will be now explored.

### 4.1. Reclaiming Security: Children’s Growing from Directing to Playfully Exploring

Findings point to the delicate ways children used the therapeutic context to restore their sense of control and feelings of reassurance, as experienced within their relationships with their parents. Most intriguing, all children included in this study utilized the music sessions to gain a sense of safety through deploying the musical experiences, first and foremost, to dictate the minute-by-minute occurrences in the session: whether by giving the parent and therapist specific musical roles, or by directing the adults in the room to hold their breath. Such behaviors reflected not only the children’s high levels of anxiety, but, more deeply, it revealed their inability to trust their central attachment figures. Various studies have shown the devastating effect of trauma on children’s levels of secure attachment [4,61], as well as on children’s extent of freedom and playfulness [62,63].

While CPP was widely proved to decrease traumatic symptoms and restore significant aspects of the child–therapist relationship [36,61,64], this study is the first to show how a CPP-informed approach in music therapy practice may contribute to rebuilding confidence, faith and security among dyads who had experienced war induced trauma.

In addition, and congruent with various acute trauma and post-trauma intervention protocols [65,66,67,68], this study emphasized the significance of establishing with clients their inner resources and exercising with them states of grounding and regulation. The current study demonstrated how child–parent and child–therapist joint music making might serve as a regulative activity, supporting clients in visiting their traumatic experiences, as well as in taking a healthy distance from the trauma at the end of an intervention. In this sense, it seems that the musical experience enabled the dyads both to reach a regulative state, which allowed them to touch on traumatic memories, and to further gain additional levels of playfulness and freedom, utilizing the musical environment.

This research’s findings conform with additional studies highlighting the significance of embracing a biopsychosocial framework when working with children who had experienced trauma [36,61,64]. The therapist’s emphasis on restoring the child’s most primary, biological and physiological sense of control, leading to an increase in both parents’ and children’s regulated state, corresponds to additional perspectives, which urge clinicians to include a comprehensive view of the child’s being [69] and on conducting a thorough assessment of the child’s history to gain all the required information possibly linked to various developmental symptoms experienced by the child [5,11]. As indicated by additional ecological models for the treatment of childhood trauma [36,61], after reaching a more regulated state, both child and parent were free to explore their environments and relationships more playfully.

### 4.2. Rehabilitating Parental Sense of Competency

A central component unearthed across various themes relates to the extensive use of the sessions as a means for rebuilding parents’ position as being competent and present. In this respect, parents were granted the opportunity to revisit their children’s moments of horror by co-creating their war narratives and connecting to parts which were inaccessible for parents and children up until that moment. CPP research advocates for the need to “speak the unspeakable”, and for supporting parents in establishing an environment that would allow tapping into the traumatic experiences [21,36,61,64]. This research further highlights different ways whereby musical experiences can assist parents in delving into such horrifying moments and have a chance to witness and acknowledge their child’s experiences, especially in situations where they could not fully be present, e.g., when the terror attack occurred. Music, and specifically songwriting, was found to provide a mediating role, allowing both the child to connect to their experience and the parent to be able to contain their child’s unbearable experiences. Moreover, engaging in a performance or finalizing a musical recording seemed to provide families with a concrete object to hold on to and relate to, an object representing their sense of healing, reconnecting, and celebrating life, rather than being caught in the deadly, frozen past.

Finally, different approaches to trauma emphasized the devastating effect of trauma on both children’s [4,36] and adult’s [65] sense of helplessness, inability, failure and passiveness. This study suggests that engaging in joint music making provides a multi-layered experience that might support parent–child dyads in becoming more present and active, and in restoring their sense of agency and competency. Through becoming playful and innovative in the musical space, parents and children were able to attend to each other’s musical requests and enjoy regulative moments of joint music making. Therefore, music therapy was found to be an important approach in the context of trauma treatment for this population.

The ongoing state of war and conflict in Israel and Palestine indicated an urgent need for therapists specialized in trauma-informed practice, particularly highlighting its adverse effect on children and adolescence [9,63]. Numerous centers for supporting citizens’ resilience (i.e., “Hosen-Centers”) are spread throughout Israel. First-aid trauma in Israel is managed by social workers who specialize in trauma informed practice [70]. This study points to possible benefits of integrating music therapy-informed practice within such services: in Israel, Palestine and anywhere that trauma endures. Most importantly, it seems that the music-based intervention was able to touch on different realms of the child’s experience: the physiological (regulation), cognitive (trauma-song narrative), emotional (sharing emotions through singing and co-music), relational (interacting musically with parents and therapist), and more. This holistic approach to children who had experienced trauma ratifies the significance of applying an ecological perspective when treating childhood trauma and further suggests the possible contribution of the arts to such models.

### 4.3. Limitations

The thematic analysis of a music therapy intervention for children and adolescents who had experienced trauma revealed promising possibilities in promoting attachment, regulation and healing. This study serves as a preliminary attempt to outline the key components of a child–parent approach to trauma within the context of music therapy. However, it faces several limitations, including the use of a relatively small sample size. Further research is required to test and consolidate applicable protocols which include music making as a prominent agent. As raised by one of the auditors of this manuscript, the data analysis also failed to capture any contra-indications for using music in therapy with individuals who experienced acute stress symptoms. More research is required to reveal what kinds of clinical music making is suitable when working with this population and what kinds of music might elevate clients’ levels of stress and anxiety rather than support them. To advance the field, future research could employ micro-analytic [52] methods to explore the relationship between verbal content in specific session periods and the level and quality of both the child’s and parent’s musical engagement. As this study was based on a qualitative framework, it lacked outcome measures and a control group. For more comprehensive recommendations on trauma-informed music therapy treatment and to develop reliable protocols, future studies should include a larger sample size and measurable outcomes, enabling generalization. Expanding the sample to include more child–parent dyads would allow for the examination of the intervention’s impact on factors such as attachment, anxiety, stress, regulation, and well-being, thereby strengthening the validity and reliability of trauma-informed, music-based interventions.

## 5. Conclusions

The literature advocates for long-term studies and interventions to mitigate the enduring effects of war on children’s development, stressing the importance of evidence-based interventions aimed at preventing and treating trauma, as well as policies that prioritize the mental well-being of children in conflict zones [10,19,36].

This study showed novel evidence for the benefits of using CPP-informed music therapy in the treatment of families who had experienced a terror attack and were displaced from their home-kibbutz for more than six months. Findings revealed that CPP-informed music therapy provided ample opportunities for children and parents to revisit their traumatic experiences, promoted parental responsiveness and supported children’s and parents’ feelings of competency, agency, coherence and stability. This study underscores the significance of families engaging in musical activities, fostering a sense of safety, acknowledging their children’s traumatic experiences, and paving the way toward rehabilitation, resilience, and playfulness.

## Figures and Tables

**Table 1:** Research Participants.

Name	Gender	Age	# Sessions
Yana	F	13	6
Rani	M	8	16
Sarit	F	10	14
Maya	F	5.5	17

## Data Availability

Data are contained within the article.

## References

[1] Michalek J., Lisi M., Binetti N., Ozkaya S., Hadfield K., Dajani R., Mareschal I. (2022). War-related Trauma Linked to Increased Sustained Attention to Threat in Children. Child. Dev..

[2] Beer B.L.E., Birnbaum J.C. (2022). Trauma-Informed Music Therapy: Theory and Practice.

[3] Catani C., Schauer E., Neuner F. (2008). Beyond Individual War Trauma: Domestic Violence against Children in Afghanistan and Sri Lanka. J. Marital. Fam. Ther..

[4] Masten A.S., Narayan A.J. (2012). Child Development in the Context of Disaster, War, and Terrorism: Pathways of Risk and Resilience. Annu. Rev. Psychol..

[5] Popham C.M., McEwen F.S., Karam E., Fayyad J., Karam G., Saab D., Moghames P., Pluess M. (2023). Predictors of Psychological Risk and Resilience among Syrian Refugee Children. Child. Psychol. Psychiatry.

[6] Hazer L., Gredebäck G. (2023). The Effects of War, Displacement, and Trauma on Child Development. Humanit. Soc. Sci. Commun..

[7] Sagi-Schwartz A. (2008). The Well Being of Children Living in Chronic War Zones: The Palestinian—Israeli Case. Int. J. Behav. Dev..

[8] Khamis V. (2019). Posttraumatic Stress Disorder and Emotion Dysregulation among Syrian Refugee Children and Adolescents Resettled in Lebanon and Jordan. Child. Abus. Negl..

[9] Pat-Horenczyk R., Achituv M., Rubenstein A.K., Khodabakhsh A., Brom D., Chemtob C. (2012). Growing Up Under Fire: Building Resilience in Young Children and Parents Exposed to Ongoing Missile Attacks. J. Child. Adol Trauma..

[10] Bryant R.A., Edwards B., Creamer M., O’Donnell M., Forbes D., Felmingham K.L., Silove D., Steel Z., Nickerson A., McFarlane A.C. (2018). The Effect of Post-Traumatic Stress Disorder on Refugees’ Parenting and Their Children’s Mental Health: A Cohort Study. Lancet Public. Health.

[11] Perry B.D. (2009). Examining Child Maltreatment Through a Neurodevelopmental Lens: Clinical Applications of the Neurosequential Model of Therapeutics. J. Loss Trauma.

[12] Shapiro J.R., Applegate J.S. (2000). Cognitive Neuroscience, Neurobiology and Affect Regulation: Implications for Clinical Social Work. Clin. Soc. Work. J..

[13] Kapel Lev-ari R., Aloni R., Ben-ari A. (2024). Understanding the Dyadic Mental Health of Refugee Parents and Children after Fleeing the 2022 Ukraine War. Psychol. Trauma Theory Res. Pract. Policy.

[14] Murphy K.M., Rodrigues K., Costigan J., Annan J. (2017). Raising Children in Conflict: An Integrative Model of Parenting in War. Peace Confl. J. Peace Psychol..

[15] Gewirtz A., Forgatch M., Wieling E. (2008). Parenting Practices as Potential Mechanisms for Child Adjustment Following Mass Trauma. J. Marital. Fam. Ther..

[16] Hoffman Y., Shrira A. (2019). Variables Connecting Parental PTSD to Offspring Successful Aging: Parent–Child Role Reversal, Secondary Traumatization, and Depressive Symptoms. Front. Psychiatry.

[17] Möllerherm J., Saile R., Wieling E., Neuner F., Catani C. (2024). Parenting in a Post-Conflict Region: Associations between Observed Maternal Parenting Practices and Maternal, Child, and Contextual Factors in Northern Uganda. Dev. Psychopathol..

[18] Khamis V. (2016). Does Parent’s Psychological Distress Mediate the Relationship between War Trauma and Psychosocial Adjustment in Children?. J. Health Psychol..

[19] Chemtob C.M., Nomura Y., Rajendran K., Yehuda R., Schwartz D., Abramovitz R. (2010). Impact of Maternal Posttraumatic Stress Disorder and Depression Following Exposure to the September 11 Attacks on Preschool Children’s Behavior. Child. Dev..

[20] Hoffnung-Assouline A., Knei-Paz C. (2024). Supervising Contact Visits: A Trauma-Informed Approach Based on Principles of Child-Parent Psychotherapy. Clin. Soc. Work J..

[21] Hagan M.J., Browne D.T., Sulik M., Ippen C.G., Bush N., Lieberman A.F. (2017). Parent and Child Trauma Symptoms During Child–Parent Psychotherapy: A Prospective Cohort Study of Dyadic Change. J. Trauma. Stress.

[22] Gordon J. (2024). Longing and Belonging: Making Mobiles in Art Therapy with Young Ukrainian Refugees. Int. J. Art Ther..

[23] Bensimon M. (2022). Mechanisms of Change in Music Therapy When Treating Adults Coping with Trauma. Trauma-Informed Music Therapy.

[24] Bensimon M. (2020). Relational Needs in Music Therapy with Trauma Victims: The Perspective of Music Therapists. Nord. J. Music. Ther..

[25] Nnanyelugo C.E., Iyendo T.O., Emmanuel N.O., Okwuowulu C., Izuchukwu John E., Apuke O.D., Gever V.C. (2023). Effect of Internet-Mediated Music Therapy Intervention on Reduction in Generalized Anxiety Disorder Symptoms among Displaced Nigerians of the Russia–Ukraine War. Psychol. Music..

[26] Abrams B. (2021). Encountering Transgenerational Trauma through Analytical Music Therapy. Nord. J. Music Ther..

[27] Wilhelmsen C., Fuhr G. (2018). Reflections on Practice. Voices.

[28] Uhlig S., Jansen E., Scherder E. (2018). “Being a Bully Isn’t Very Cool…”: Rap & Sing Music Therapy for Enhanced Emotional Self-Regulation in an Adolescent School Setting—A Randomized Controlled Trial. Psychol. Music..

[29] Moore K.S., Hanson-Abromeit D. (2023). Feasibility of the Musical Contour Regulation Facilitation (MCRF) Intervention to Promote Emotion Regulation for under-Resourced Preschoolers: Examining Intervention Intensity. Arts Psychother..

[30] Zhang M., Ding Y., Zhang J., Jiang X., Xu N., Zhang L., Yu W. (2022). Effect of Group Impromptu Music Therapy on Emotional Regulation and Depressive Symptoms of College Students: A Randomized Controlled Study. Front. Psychol..

[31] Sena Moore K., Hanson-Abromeit D. (2015). Theory-Guided Therapeutic Function of Music to Facilitate Emotion Regulation Development in Preschool-Aged Children. Front. Hum. Neurosci..

[32] Malloch S., Trevarthen C. (2009). Communicative Musicality: Exploring the Basis of Human Companionship.

[33] Chinchaipong P. (2022). Association of Post-Traumatic Stress Disorder (PTSD) in Children and HPA Axis Regulation. Int. J. Sci. Healthc. Res..

[34] Russo J.E., Dhruve D.M., Oliveros A.D. (2023). Childhood Trauma and PTSD Symptoms: Disentangling the Roles of Emotion Regulation and Distress Tolerance. Res. Child. Adolesc. Psychopathol..

[35] Malloch S.N. (1999). Mothers and Infants and Communicative Musicality. Music. Sci..

[36] Lieberman A.F., Van Horn P. (2011). Psychotherapy with Infants and Young Children: Repairing the Effects of Stress and Trauma on Early Attachment, Paperback ed..

[37] Colegrove V.M., Havighurst S.S., Kehoe C.E. (2019). Emotion Regulation during Conflict Interaction after a Systemic Music Intervention: Understanding Changes for Parents with a Trauma History and Their Adolescent. Nord. J. Music. Ther..

[38] Chifa M., Hadar T., Politimou N., Reynolds G., Franco F. (2021). The Soundscape of Neonatal Intensive Care: A Mixed-Methods Study of the Parents’ Experience. Children.

[39] Loewy J. (2015). NICU Music Therapy: Song of Kin as Critical Lullaby in Research and Practice. Ann. N. Y. Acad. Sci..

[40] Loewy J.V., Rossetti A., Telsey A., Dassler A.M. (2021). Assessing & Treating Trauma: Music Psychotherapy for Parents of Neonates. Music. Med..

[41] Saada B., Coombes E. (2020). Music Therapy in the Occupied Palestinian Territories: An Overview and Some Perspectives on Dementia and End-of-Life Care. Approaches Interdiscip. J. Music. Ther..

[42] Enge K.E.A., Stige B. (2022). Musical Pathways to the Peer Community: A Collective Case Study of Refugee Children’s Use of Music Therapy. Nord. J. Music. Ther..

[43] Mallon T., Hoog Antink M. (2021). The Sound of Lost Homes—Introducing the COVER Model—Theoretical Framework and Practical Insight into Music Therapy with Refugees and Asylum Seekers. Voices.

[44] Storsve V., Westbye I.A., Ruud E. (2010). Hope and Recognition: A Music Project among Youth in a Palestinian Refugee Camp. Voices.

[45] Bottema-Beutel K., Kapp S.K., Lester J.N., Sasson N.J., Hand B.N. (2021). Avoiding Ableist Language: Suggestions for Autism Researchers. Autism Adulthood.

[46] Hadar T. (2022). “What Sound Does a Cat Make in Cantonese?”: Advocating for Lingual Plurality in Music Therapy Settings. Voices.

[47] Ferrara L. (1984). Phenomenology as a Tool for Musical Analysis. Music. Q..

[48] Smith J.A., Larkin M., Flowers P. (2008). Interpretative Phenomenological Analysis: Theory, Method and Research.

[49] Lee J., McFerran K.S. (2015). Applying Interpretative Phenomenological Analysis to Video Data in Music Therapy. Qual. Res. Psychol..

[50] Hadar T., Amir D. (2021). Intimacy, Mutuality & Negotiations: Dialogic Moments in Joint Improvisation. Nord. J. Music. Ther..

[51] Hadar T., Amir D. (2018). Discovering the Flute’s Voice: On the Relation of Flutist Music Therapists to Their Primary Instrument. Nord. J. Music. Ther..

[52] Wosch T., Wigram T., Wheeler B.L. (2007). Microanalysis in Music Therapy: Methods, Techniques and Applications for Clinicians, Researchers, Educators and Students.

[53] Hakvoort L., Tönjes D. (2023). Music-Mechanisms at the Core of Music Therapy: Towards a Format for a Description of Music Therapy Micro-Interventions. Nord. J. Music. Ther..

[54] Corbin J.M., Strauss A.L. (2015). Basics of Qualitative Research: Techniques and Procedures for Developing Grounded Theory.

[55] Charmaz K. (2006). Constructing Grounded Theory: A Practical Guide through Qualitative Analysis.

[56] Charmaz K. (1996). Rethinking Methods in Psychology. The Search for Meanings-Grounded Theory.

[57] Wheeler B.L., Murphy K.M. (2016). Music Therapy Research.

[58] Denzin N.K., Lincoln Y.S. (2009). The Sage Handbook of Qualitative Research.

[59] Bruscia K.E., Bruscia K.E. (1987). Improvisational Models of Music Therapy.

[60] Hadar T., Aigen K. (2024). Music, Time, and Self: A Time Model for Nordoff-Robbins Clinical Improvisation.

[61] Lieberman A.F., Ghosh Ippen C., Van Horn P. (2006). Child-Parent Psychotherapy: 6-Month Follow-up of a Randomized Controlled Trial. J. Am. Acad. Child. Adolesc. Psychiatry.

[62] Chazan S., Cohen E. (2010). Adaptive and Defensive Strategies in Post-Traumatic Play of Young Children Exposed to Violent Attacks. J. Child Psychother..

[63] Barbanel L., Sternberg R.J. (2006). Psychological Interventions in Times of Crisis.

[64] Sullivan A.D.W., Merrill S.M., Konwar C., Coccia M., Rivera L., MacIsaac J.L., Lieberman A.F., Kobor M.S., Bush N.R. (2024). Intervening after Trauma: Child–Parent Psychotherapy Treatment Is Associated with Lower Pediatric Epigenetic Age Acceleration. Psychol. Sci..

[65] Farchi M., Hirsch-Gornemann M.B., Whiteson A., Gidron Y. (2018). The SIX Cs Model for Immediate Cognitive Psychological First Aid: From Helplessness to Active Efficient Coping. Int. J. Emerg. Ment. Health.

[66] Shapiro E., Laub B. (2015). Early EMDR Intervention Following a Community Critical Incident: A Randomized Clinical Trial. J. EMDR Prac. Res..

[67] Zemestani M., Mohammed A.F., Ismail A.A., Vujanovic A.A. (2022). A Pilot Randomized Clinical Trial of a Novel, Culturally Adapted, Trauma-Focused Cognitive-Behavioral Intervention for War-Related PTSD in Iraqi Women. Behav. Ther..

[68] Tissue A., Specker P., Hoffman J., Uppal S., Cloitre M., Neuner F., O’Donnell M., Nickerson A. (2023). Skills Training in Affective and Interpersonal Regulation for Refugees Integrated with Narrative Exposure Therapy: A Case Study on the Treatment of PTSD and Emotion Dysregulation for Refugees and Asylum-Seekers. Clin. Case Stud..

[69] Berzoff J., Drisko J. (2015). What Clinical Social Workers Need to Know: Bio-Psycho-Social Knowledge and Skills for the Twenty First Century. Clin. Soc. Work. J..

[70] Cohen M., Gagin R., Peled-Avram M. (2006). Multiple Terrorist Attacks: Compassion Fatigue in Israeli Social Workers. Traumatology.

